# Application of Electronic tongue and HPLC in rapid determination of functional triterpenes and origins of *Ganoderma lucidum*

**DOI:** 10.3389/fnut.2024.1446956

**Published:** 2024-10-21

**Authors:** Jing Tian, Jinfeng Wei, Yuxin Liu, Changqin Li, Changyang Ma, Wenyi Kang

**Affiliations:** ^1^National R&D Center for Edible Fungus Processing Technology, Henan University, Kaifeng, China; ^2^The First Affiliated Hospital of Henan University, Kaifeng, China; ^3^Huaihe Hospital of Henan University, Kaifeng, China; ^4^College of Agriculture, Henan University, Kaifeng, China; ^5^Functional Food Engineering Technology Research Center, Kaifeng, China; ^6^Joint International Research Laboratory of Food and Medicine Resource Function, Kaifeng, China

**Keywords:** *Ganoderma lucidum*, Electronic tongue, HPLC, triterpenes, origins

## Abstract

**Introduction:**

*Ganoderma lucidum* is one well known functional food resource. The triterpenes, such as Ganoderic acid A and Ganoderic acid D, as well as the sensory characteristics could reflect the quality of *G. lucidum*.

**Methods:**

In order to find rapid methods to evaluate the *Ganoderma lucidum* from different origins, Electronic tongue and High performance liquid chromatography (HPLC) were used in this paper.

**Results:**

The Electronic tongue results combined with PCA and LDA analysis showed that the taste of different batches of *G. lucidum* from the same producing area was similar, but quite different from different producing areas. The overall taste of *G. lucidum* from Anhui was obviously different from that from Shandong and Sichuan. Meanwhile, the concentrations of two main triterpenes of *G. lucidum*, Ganoderic acid A and Ganoderic acid D were detected by using HPLC, and the variability of different origins were consistent with that from Electronic tongue. Moreover, the triterpenoid acids content in *G. lucidum* from Shandong was about 1.04 mg/g, which is the highest of the three origins, followed by Sichuan and Anhui.

**Discussion:**

Both the Electronic tongue and HPLC could efficiently distinguish the different origins of *G. lucidum* from taste property or content of key triterpenes, and provide new technical support for the quality evaluation of *G. lucidum*.

## 1 Introduction

*Ganoderma lucidum* is usually known as “*Glossy Ganoderma grass*,” “*Ruicao*,” “*Revival Grass*,” which is also named as “*Ten Thousand Year mushroom*” in Japan (1, 2). *G. lucidum* contains abundant bioactive compounds, such as triterpenes, polysaccharides, sterols, etc. (3, 4), which can effectively inhibit tumors, improve immunity, improve sleep, etc. (2, 5–7) as well as prolonging lifespan (8). With the people’s pursuit of high-level living and in-depth knowledge about *G. lucidum*, the demand for this functional food resource and derivatives has also sharply increased.

Ganoderic acid A and Ganoderic acid D are reported as two representative bioactive components of *G. lucidum* (9). Many researches have shown that Ganoderic acid A could inhibit tumor growth (10), protect nerve cells (11), combat depression (12), regulate lipid metabolism (13), improve osteoarthritis (14), promote myelin sheath regeneration (15), etc. Ganoderic acid D could prevent oxidative stress-induced aging by regulating the CaM/CaMKll/NRF2 signaling pathway in stem cells (16). This compound can also be used for the treatment of esophageal squamous cell carcinoma through promoting ESCC cell apoptosis and autophagy cell death (17). In our previous study, Ganoderic acid A and Ganoderic acid D were found to be the key components with immunomodulatory activity, and had obvious enhanced immune activity (9).

For quality management of *G. lucidum*, many technologies were used. According to relevant literature reports, HPLC is a commonly used analytical technique for component content determination (18), with strong operability, high resolution, wide applicability, and high safety (19, 20). The standard HPLC method for determination of Ganoderic acid A and Ganoderic acid D is available. Moreover, these critical triterpenoid acids are not only the bioactive compounds of *G. lucidum*, but also strongly associated with the taste of this material (21), indicating that it would be possible to detect the taste properties to evaluate the quality of *G. lucidum*. However, since the strong subjectivity of artificial taste and the weak stability, repeatability, and reliability of human sensor organ, sensor analysis method was greatly limited in the quality control on functional materials, until the application of electronic tongues (22, 23). Electronic tongue technology can simulate human perception of taste through sensor array, so as to detect non-volatile compounds in sample solution (24). The characteristics of electronic tongue technology enable the detection of a specific type of substance rapidly, while HPLC method allows for the identification of particular components. The combination of these two techniques is more conducive to comprehensive analysis of specific substances and there results could be confirmed mutually. In the research of Zabada et al. (25), Electronic tongue technology and HPLC combined with PLSR method were used to detect the concentration of 2-phenylethanol and glucose in yeast culture medium. This method can rapid monitor the yeast fermentation processes with some specific analytes.

On this basis, this study would use Electronic tongue technology combined with chemometrics to identify the origins of *G. lucidum* simply, accurately and quickly. HPLC technology was also used to determine the content of Ganoderic acid A and Ganoderic acid D in *G. lucidum*, and study the differences in the content of triterpenoids in *G. lucidum* from different regions. Partial least squares regression (PLSR) model was adopted to study the correlation between sensory quality parameters and characteristic flavor substances of *G. lucidum*, in order to provide scientific basis for quality control and reasonable application of *G. lucidum*.

## 2 Materials and methods

### 2.1 Materials

#### 2.1.1 Samples

A total of 20 dried samples were collected from the three major producing areas of *G. lucidum* with large yield and good quality, including the provinces of Shandong, Anhui and Sichuan in China and labelled as L1 to W20. L1 ~ L7 samples were from Shandong province, S8 ~ S12 samples were from Sichuan province, and W13 ~ W20 samples were from Anhui province.

#### 2.1.2 Instruments and equipment

The LC-20AT HPLC system was pursued from Shimadzu, Kyoto in Japan; 2,908,008 Electronic tongues with 6 sensors (C1-C6) was produced by Shanghai Ruisun Intelligent Technology Co., Ltd. in China, and the Unscrambler X10.4 statistical analysis software was developed by CAMO Inc. in Norway.

Acetonitrile and methanol were chromatographic grade, while Glacial acetic acid was analytical grade. The standard Ganoderic acid A (JOT-10947) and Ganoderic acid D (JOT-11170) were purchased from Chengdu Pufeide Biotechnology Co., Ltd. in China.

### 2.2 Experimental methods

#### 2.2.1 Preparation of test solution

After grinding of *G. lucidum* into powder, 2.00 g of each sample powder was accurately weighed, and dissolved in 20 mL methanol. Ultrasonic extraction was performed for 1 h, followed by centrifugation for 10 min. Supernatant was taken and filtered through 0.22 μm microporous filter membrane. The subsequent filtrate was collected as the test solution.

#### 2.2.2 Electron tongue detection

At first, the 6 electronic tongue sensors should be activated and preheated for 30 min. And then, clean the sensors with distilled water for 120 s and put them into the test solution for 180 s to test the sample. Each sample was tested 3 times in parallel, and take their average value as the test result for analysis.

#### 2.2.3 Preparation of standard solution

Weigh the reference materials of Ganoderic acid A and Ganoderic acid D accurately and prepare the two standard solutions at the concentration of 2 mg/mL with methanol. Then, the two standard solutions were diluted into a series of solutions with concentration gradient.

#### 2.2.4 Detection of compounds by HPLC

##### 2.2.4.1 HPLC chromatographic conditions

In the HPLC analysis, chromatographic column was the SHIMADZU Shim-pack GIST C18 (4.6 mm × 250 mm, 5 μm), detection wavelength was set at 254 nm, column temperature was 30°C, injection volume was 20 μL, and the flow rate was 0.8 mL/min. The mobile phases were acetonitrile (A) and 0.2% acetic acid aqueous solution (B), according to the gradient elution program shown in Table 1.

**Table 1:** The elution program.

Elution time /min	A/% (Acetonitrile)	B/% (0.2% Acetic acid aqueous solution)
0–15	5–28	95–72
15–30	28–30	72–70
30–60	30–39	70–61
60–70	39–54	61–46
70–88	54–75	46–25

**Figure 1:**
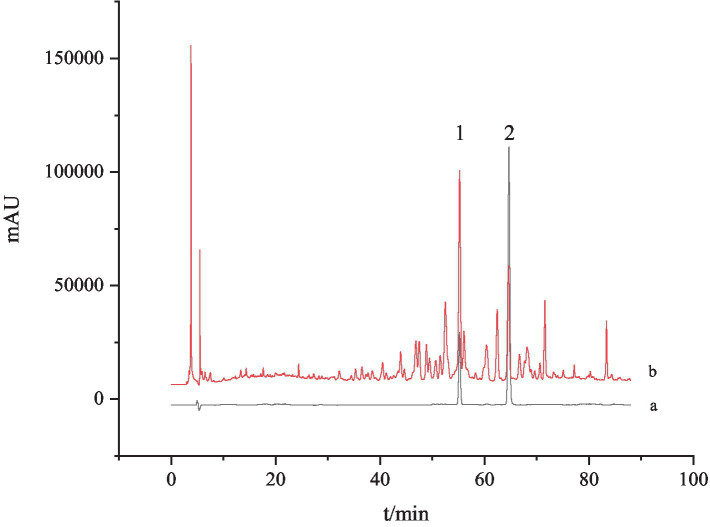
HPLC chromatogram of *G. lucidum* and standard solution. a: The standard solution and b: the test solution; 1: Ganoderic acid A and 2: Ganoderic acid D.

According to the above chromatographic conditions, the HPLC chromatogram was recorded as Figure 1. It could be found that the peaks of Ganoderic acid A and Ganoderic acid D were noticeable in *G. lucidum* samples, and the chromatographic peak separation was good, without obvious interference by other components.

##### 2.2.4.2 Methodological investigation

*Investigation of linear relationship*. The standard solution of Ganoderic acid A and Ganoderic acid D were diluted to 6 gradient concentrations and performed according to the chromatographic conditions under “*2.2.4*.” Take the injection mass concentration (*X*, mg/mL) as the argument and peak area (*Y*) as the dependent variable, and obtain the linear regression equations shown in Table 2.

**Table 2:** Regression equations and linear relationships.

Standard	Regression equation	*R* ^2^	Linear range (mg/mL)
Ganoderma acid A	*Y* = 20,506,801.6892 *X* − 4,439.7779	0.9997	0.001 ~ 0.150
Ganoderma acid D	*Y* = 21,102,145.2516 *X* − 56,065.5373	0.9993	0.001 ~ 0.200

**Figure 2:**
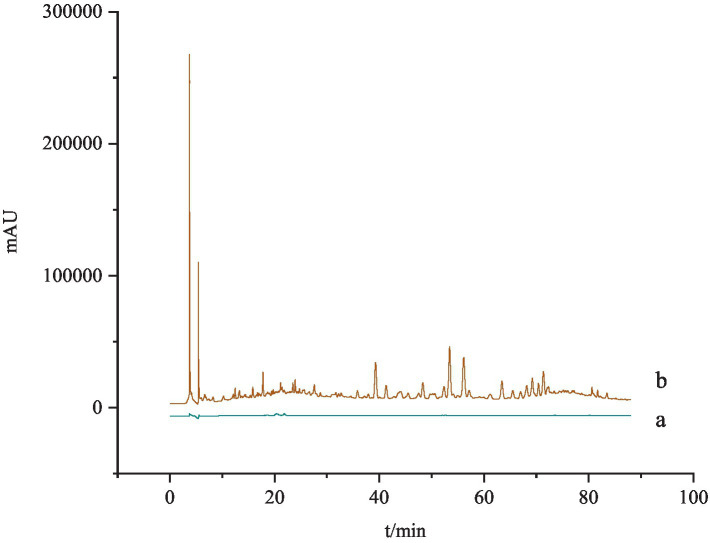
The specificity test of HPLC chromatogram for Ganoderma acid A and Ganoderma acid D. a: The blank solution; b: the test solution.

*Specificity test*. One part of blank solvent (methanol) and one part of *G. lucidum* test solution was taken and determined according to the chromatographic conditions under “2.2.4.” It was found that the blank solvent did not interfere with the determination, indicating that the method has well specificity (Figure 2).

*Precision test*. The mixed reference solution was precisely measured, and the peak area was measured by 6 consecutive samples according to the above chromatographic conditions. The RSD values of Ganoderic acid A and Ganoderic acid D were 0.13 and 0.11%, respectively, and RSD values of relative peak area were 1.25 and 1.05%, respectively, indicating good precision of the HPLC instrument.

*Repeatability test.* 6 samples were accurately weighed, the test solution was prepared according to the method under “*2.2.1*,” and its peak area was determined according to the above chromatographic conditions. RSD of Ganoderic acid A and Ganoderic acid D were 0.24 and 0.21%, respectively, and RSD of relative peak area were 1.18 and 1.20%, respectively. The reproducibility of the method was good.

*Stability test.* The same sample solution was precisely measured, and the peak area of the target component was measured at 0, 2, 6, 12, 18 and 24 h according to the above chromatographic conditions. The RSD values of the relative retention time of Ganoderic acid A and Ganoderic acid D were 0.28 and 0.25%, and the RSD values of the relative peak area were 1.45 and 1.74%, respectively, indicating that the test solution was basically stable within 24 h.

*Recovery rate test.* 6 samples of the same test product with known content were accurately weighed, appropriate amount of standard solution was added, and treated according to the method under “2.2.1.” Peak area was determined according to the above chromatographic conditions. After calculation, the average recovery rates of Ganoderic acid A and Ganoderic acid D were 100.03 and 100.44%, respectively, with RSDs of 0.92 and 0.54%.

### 2.3 Data processing

The content of different batches of *G. lucidum* were statistically analyzed and plotted using Microsoft Excel 2010 and Origin 2019b 64Bit software. The Unscrambler X 10.4 software was used for PCA and LDA analysis, and PLSR model was adopted to analyze the correlation between sensory data of *G. lucidum* and the content of flavoring substances.

## 3 Results and analysis

### 3.1 The sensory characteristics of *G. lucidum* from different regions based on electronic tongue

Different batches of *G. lucidum* solution were immersed in the Electronic tongue sensor for determination, and the sensory data were shown in Table 3.

**Table 3:** Sensory data of *G. lucidum* by Electronic tongue.

Sample	C1	C2	C3	C4	C5	C6
L1	−7.9E-06	−7.3E-06	−7E-06	−9.4E-06	−8.1E-06	−5.1E-06
L2	−7.7E-06	−7.1E-06	−6.8E-06	−9.4E-06	−8E-06	−5.6E-06
L3	−7.5E-06	−9E-06	−7.3E-06	−8.3E-06	−9E-06	−9.6E-06
L4	-6E-06	−9.7E-06	−7.8E-06	−8.1E-06	−9.3E-06	−1.1E-05
L5	−7.7E-06	−9.5E-06	−7.3E-06	−8.5E-06	−8.8E-06	−7.3E-06
L6	−6.9E-06	−7.7E-06	−4.6E-06	−7.2E-06	−8.9E-06	−8.5E-06
L7	−5.9E-06	−7.1E-06	−8.7E-06	−7.1E-06	−7.4E-06	−7.6E-06
S8	−6.4E-06	−5.7E-06	−5.6E-06	−7.8E-06	−6.4E-06	−5.2E-06
S9	−5.6E-06	−6.3E-06	−4.2E-06	−5.6E-06	−6.4E-06	−4.3E-06
S10	−5.9E-06	−6.6E-06	−6.9E-06	−6.5E-06	−6.7E-06	−4.5E-06
S11	−5.3E-06	−5.6E-06	−3.2E-06	−5.2E-06	−6.9E-06	−5.6E-06
S12	−5.7E-06	−6.6E-06	−4.5E-06	−5.9E-06	−7.1E-06	-4E-06
W13	−5.2E-06	−6.8E-06	−7.4E-06	−6.4E-06	−1.1E-05	7.42E-07
W14	−5.6E-06	−5.9E-06	−3.1E-06	−5.1E-06	−6.1E-06	−2.7E-06
W15	−5.5E-06	−5.7E-06	−3.6E-06	−5.3E-06	−6.1E-06	−2.9E-06
W16	−5.4E-06	−5.4E-06	−3.3E-06	−5.2E-06	−6.3E-06	−1.5E-06
W17	−5.4E-06	−5.5E-06	−3.5E-06	−5.3E-06	−6.2E-06	−3.1E-06
W18	−5.4E-06	−5.8E-06	−2.9E-06	−5.4E-06	−6.8E-06	−3.6E-06
W19	−5.8E-06	−6.2E-06	−3E-06	−5.4E-06	-6E-06	−3.2E-06
W20	−5.3E-06	−5.6E-06	−3.7E-06	−5.2E-06	−6.4E-06	−2.5E-06

**Figure 3:**
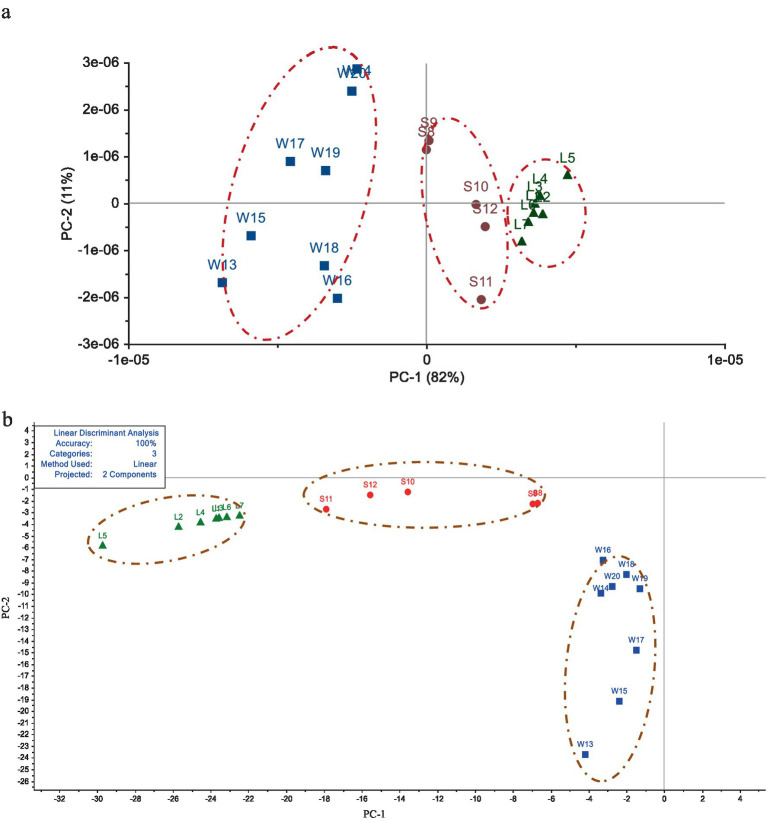
Analysis of *G. lucidum* sensory characteristics among different origins by PCA and LDA methods. (a) The result based on PCA analysis. (b) The result based on LDA distribution.

The PCA and LDA analysis of Electronic tongue taste data of the 20 batches of *G. lucidum* were shown in Figure 3. In Figure 3a, the first principal component (PC-1) accounted for 82% of the overall information content, and the second principal component (PC-2) accounted for 11%. The total contribution rates of PC-1 and PC-2 were 93%, which could basically represent all the information of the *G. lucidum* samples. The specimens from different producing areas were distributed in different areas without cross coincide between each other, indicating that there were obvious differences in sensory quality of *G. lucidum* exponents from its’ productions. From Figure 3a, it could also be perceived that the spatial distribution sites of the swatchs from Sichuan and Shandong regions were relatively proximity, but farther away from Anhui. This showed that the sensory organ of *G. lucidum* from Sichuan and Shandong was relatively close, but significant discrepancy with *G. lucidum* from Anhui. As LDA results the shown in Figure 3b, the accuracy of the linear discriminant analysis model was 100%. The first two principal components by LDA algorithm could cluster 20 batches of *G. lucidum* samples into 3 categories, which could well distinguish *G. lucidum* from different origins. Meanwhile, the samples from Sichuan province located in medial position, which was similar to the results of PCA analysis. In summary, the Electronic tongue technology combined with PCA or LDA method could effectively distinguish the *G. lucidum* from different regions.

### 3.2 Content determination and methodology investigation by HPLC

**Figure 4:**
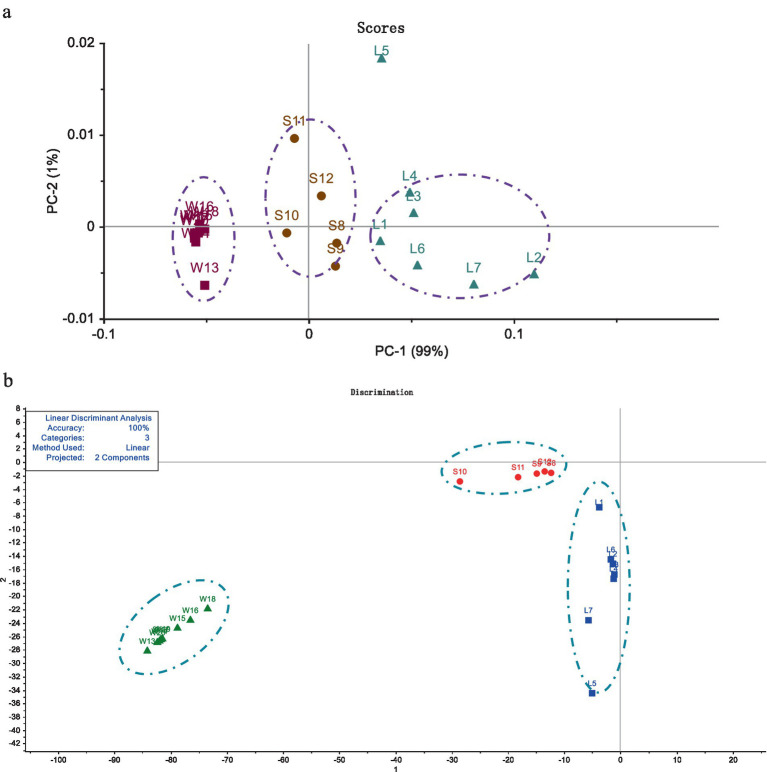
Analysis of Ganoderma acid A and Ganoderma acid D among different origins by PCA and LDA methods. (a) The result of PCA analysis. (b) The result of LDA distribution.

The contents of Ganoderic acid A and Ganoderic acid D in 20 batches of *G. lucidum* were shown in Table 4. From this, among the three grow areas of *G. lucidum*, the content of Ganoderic acid A and Ganoderic acid D in *G. lucidum* produced in Shandong is the highest, with an average of 1.04 mg/g, which is about 2 times that of Sichuan *G. lucidum* and 5 times of that from Anhui province. Through PCA and LDA analysis, the two PCs of *G. lucidum* samples were shown visually in Figure 4. It could be intuitively observed that the content of triterpenoid acids varies significantly among different regions. The boundaries among the samples from different regions without crossing each other, indicating the two triterpenoid acids could distinguish the *G. lucidum* from different regions. Compared with PCA analysis (Figure 4a), in LDA analysis results (Figure 4b), the scattered points of *G. lucidum* from the same origin were more concentrated, while those from different origins have longer scatter distances. The results of HPLC were consistent with those of Electronic tongue, indicating that Electronic tongue would be alternative methods to replace HPLC to distinguish the *G. lucidum* samples from different places, accurately and quickly. And sense organ could mirror the markers of *G. lucidum*, to a certain extend.

**Table 4:** The contents of Ganoderma acid A and Ganoderma acid D in 20 batches of *G. lucidum.*

Sample number	Concentration /(mg/g)	
	Ganoderma acid A	Ganoderma acid D
L1	0.8087 ± 0.04	0.4635 ± 0.03
L2	1.4571 ± 0.03	0.8424 ± 0.05
L3	0.9661 ± 0.03	0.5120 ± 1.85
L4	0.9619 ± 0.27	0.4844 ± 0.07
L5	0.9083 ± 0.60	0.2917 ± 0.04
L6	0.9574 ± 0.04	0.5706 ± 0.87
L7	1.1942 ± 0.04	0.7189 ± 0.08
S8	0.6233 ± 0.05	0.3686 ± 0.05
S9	0.6027 ± 0.05	0.3859 ± 0.05
S10	0.4115 ± 0.01	0.2442 ± 0.10
S11	0.4907 ± 0.05	0.1706 ± 0.33
S12	0.5768 ± 0.04	0.2862 ± 0.04
W13	0.1294 ± 0.10	0.0943 ± 0.09
W14	0.0145 ± 0.02	0.0489 ± 0.07
W15	0.0286 ± 0.06	0.0450 ± 0.02
W16	0.0364 ± 0.04	0.0372 ± 0.06
W17	0.0119 ± 0.08	0.0384 ± 0.05
W18	0.0588 ± 0.06	0.0554 ± 0.02
W19	0.0115 ± 0.07	0.0352 ± 0.05
W20	0.0093 ± 0.05	0.0408 ± 0.04

### 3.3 Correlation analysis of triterpenoid acids and sensory properties of *G. lucidum*

**Figure 5:**
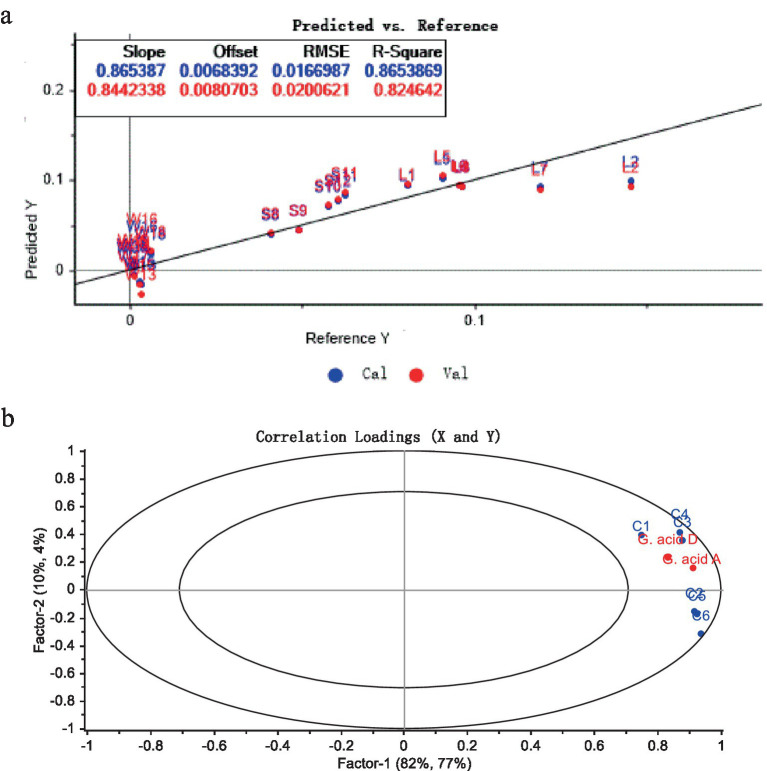
PLSR correlation analysis of Ganodermic acid A and D with sensory quality characteristics of *G. lucidum*. (a) Linear graph of model predicted value vs reference data. (b) Related load distribution diagram.

The correlation between the sensory properties of *G. lucidum* by electronic tongue and the content of 2 key triterpenoids by HPLC was analyzed by PLSR. Multi-independent and multi-dependent variable analysis was conducted with the flavor characteristic data as X variables and the contents of Ganoderic acid A and Ganoderic acid D as Y variables, and the results were shown in Figure 5. Figure 5a was the comparison chart of PLSR model prediction. Four indexes including Slope, Offset, RMSE and R-Square were used to evaluate the performance of the model. Generally, the smaller of the Offset and RMSE and the closer distance of the Slope and R-Square to 1, the better the prediction effect of the model (26). In this model, blue was the correction set and red was the verification set. Slope and R-Square were greater than 0.80, Offset and RMSE are close to zero, sample points were scattered around the straight line as y = x. The less fluctuation range between predicted and measured values declared that the model has high prognosis accuracy.

The correlation load diagram between the triterpenoid components of *G. lucidum* and their sensory characteristics of taste were shown in Figure 5b. The two principal components (Factor-1 and Factor-2) of the established PLSR regression model explained 95% of the cross-validation variance, expressing that the model could successfully reflect the overall information of *G. lucidum* samples. The two ellipses in the figure ertr R^2^ = 0.5 (small ellipse) and R^2^ = 1.0 (large ellipse), respectively, representing 50 and 100% interpretation variance (27). In Figure 5b, all variables fell between two ellipses, indicated that these variables could be commendably guided by the PLSR mold. Ganoderic acid A, Ganoderic acid D and 6 electronic tongue sensors were located on the starboard of the biplot, illustrated that Ganoderic acid A, Ganoderic acid D were strikingly positively correlated with the six sensor indicators. The results declared that Ganoderma A and Ganoderma D might proffer much to the taste of *G. lucidum*. This results also supported that, the electronic tongue technology could preferably identify the taste character of *G. lucidum*, and could be one rapid method for the origin identification and quality control of *G. lucidum*.

## 4 Discussion

The Electronic tongue test results combined with PCA and LDA analysis for origin identification of *G. lucidum* were basically consistent with the HPLC. All the samples were clustered into 3 categories according origins without overlap. And that results displayed that the taste of different batches of *G. lucidum* in the same origin was similar, and the difference of *G. lucidum* in different origin was obvious. The overall taste of *G. lucidum* produced in Anhui Province is obviously different from that in Shandong Province and Sichuan Province. According to the results of Ganoderic acid A and Ganoderic acid D in *G. lucidum* by using HPLC, *G. lucidum* in the three provinces had obviously difference in content of Ganoderic acid ingredients. This might be closely linked with many factors such as genetic characteristics, growth and development stage, temperature, medium and geographical and climatic conditions of *G. lucidum* (28). Within the two key components of *G. lucidum*, the content of Ganoderic acid A was generally higher than that of Ganoderic acid D, which was consistent with the analysis results of the triterpenoid content of *G. lucidum* by HPIC method by Liu et al. (29). Moreover, results of sensory properties presented by Electronic tongue with PLSR were also closely associated with the triterpenoid acids results by HPLC. The absolute coefficients R^2^ of the correction set and validation set were 0.8654 and 0.8246 respectively, showing a relatively good linear relationship and significant positive correlation. This reflected the Electronic tongue could be used for the quality control of this product, to a certain extent.

Based on the results by Electronic tongue, the overall taste of *G. lucidum* from different origins were relatively different, especially from Anhui. Similar results were get based on the HPLC. The distribution map based on the content of *G. lucidum* triterpenoid acids showed the close distance between the samples from Sichuan and Shandong, but relative far between Anhui and the other two producing areas. Specifically, the contents of two triterpenoid acids in *G. lucidum* were the highest in Shandong province (1.04 mg/g), followed by Sichuan province and Anhui province. Within the two triterpenoids among samples from the 3 provinces, the difference of Ganoderic acid A in *G. lucidum* were much marked than that of Ganoderic acid D. In addition, the content of Ganoderma A in the *G. lucidum* was generally higher than that of Ganoderma D, which was consistent with the research by Liu et al. (29) by using HPIC method.

## 5 Conclusion

In summary, it was feasible to quickly determine *G. lucidum* from different regions based on Electronic tongue technology and HPLC method. Both the methods could quickly and effectively determine the Ganoderma A and Ganoderma D of *G. lucidum*. The electronic tongue technology could easily and quickly distinguish the flavor characteristics of *G. lucidum* from different sources, and provided new technical support for its origin identification and flavor research, and supply the theoretical support for the development of *G. lucidum* and related products.

## Data Availability

The raw data supporting the conclusions of this article will be made available by the authors, without undue reservation.
